# Cytokine Response after Stimulation with Key Commensal Bacteria Differ in Post-Infectious Irritable Bowel Syndrome (PI-IBS) Patients Compared to Healthy Controls

**DOI:** 10.1371/journal.pone.0134836

**Published:** 2015-09-14

**Authors:** Johanna Sundin, Ignacio Rangel, Dirk Repsilber, Robert-Jan Brummer

**Affiliations:** School of Health and Medical Sciences, Faculty of Medicine and Health, Örebro University, Örebro, Sweden; University of California, Los Angeles, UNITED STATES

## Abstract

**Background:**

Microbial dysbiosis and prolonged immune activation resulting in low-grade inflammation and intestinal barrier dysfunction have been suggested to be underlying causes of post-infectious irritable bowel syndrome (PI-IBS). The aim of this study was to evaluate the difference in cytokine response between mucosal specimens of PI-IBS patients and healthy controls (HC) after *ex vivo* stimulation with key anaerobic bacteria.

**Methods:**

Colonic biopsies from 11 PI-IBS patients and 10 HC were stimulated *ex vivo* with the commensal bacteria *Bacteroides ovatus*, *Ruminococcus gnavus*, *Akkermansia muciniphila*, *Subdoligranulum variabile and Eubacterium limosum*, respectively. The cytokine release (IL-1β, IL-2, IL-8, IL-10, IL-13, IL-17, TNF-α and IFN-γ) in stimulation supernatants was analyzed using the LUMINEX assay. Comparison of cytokine release between PI-IBS patients and healthy controls was performed taking both unstimulated and bacterially stimulated mucosal specimens into account.

**Key Results:**

IL-13 release from mucosal specimens without bacterial stimulation was significantly lower in PI-IBS patients compared to HC *(p < 0*.*05)*. After stimulation with *Subdoligranulum variabile*, IL-1β release from PI-IBS patients was significantly increased compared to HC *(p < 0*.*05)*. Stimulation with *Eubacterium limosum* resulted in a significantly decreased IL-10 release in HC compared to PI-IBS patients *(p < 0*.*05)* and a tendency to decreased IL-13 release in HC compared to PI-IBS patients *(p = 0*.*07)*.

**Conclusions & Inferences:**

PI-IBS patients differ from HC with regard to cytokine release *ex vivo* after stimulation with selected commensal bacteria. Hence, our results support that the pathogenesis of PI-IBS comprises an altered immune response against commensal gut microbes.

## Introduction

Irritable bowel syndrome (IBS) is a functional gastrointestinal disorder characterized by chronic abdominal symptoms, including discomfort, pain and altered bowel habits. Patients are diagnosed by the ROME criteria and histological exclusion of other gut diseases. Post-infectious irritable bowel syndrome patients (PI-IBS) show the onset of disease after an episode of acute gastroenteritis with diarrhea and/or vomiting [1]. Even though the pathophysiology of IBS and PI-IBS is still not clarified, prolonged immune activation resulting in microbial dysbiosis [2–4], low-grade inflammation [5, 6], and intestinal barrier dysfunction [7] has been suggested to be underlying causes. However, the majority of studies that show alterations of the intestinal immune system and/or microbiota in IBS patients are strictly descriptive, which limits the elucidation of a temporal association between putative causes and pathophysiological effects. The intestinal barrier is composed of a single layer of epithelial cells that produce an antigen-specific immune response to intestinal microbes, which contributes to the maintenance of intestinal homeostasis and protects the host from pathogenic bacteria [8]. However, immune activation also induces cytokine release that may cause damage to the epithelial barrier resulting in influx of bacteria and antigens into the mucosa [9]. Mucosal immune activation has been associated with deterioration of the integrity of the epithelial barrier and intestinal dysfunction in IBS [10, 11].

Based on previous results of alterations in the mucosal adaptive immune system [6], eight cytokines were selected in this study representing both pro- (IL-2, IL-1β, IL-8, TNF-α, IFN-γ and IL-17) and/or anti-inflammatory (IL-2, IL-10 and IL-13) cytokines. Biopsies from PI-IBS patients, as well as healthy controls, were stimulated *ex vivo* with key anaerobic bacterial strains. These strains were selected on the basis of previous studies on differences in relative abundance of bacterial genera or strains in IBS patients compared to healthy controls (i.e. *Bacteroides ovatus*, *Ruminococcus gnavus*, *Akkermansia muciniphila* and *Eubacterium limosum)* [4], as well as on basis of their specific functionality, such as fermentation of polysaccharides (i.e. *B*. *ovatus*) [12], mucin-degrading properties (i.e. *R*. *gnavus* and *A*. *muciniphila*) [13] [14] and butyrate-production (i.e. *S*. *variabile* and *E*. *limosum*) [15, 16].

The aim of this study was to evaluate the possible difference between PI-IBS patients and healthy controls regarding the immune response to *ex vivo* stimulation of colonic mucosal specimens with key anaerobic bacteria. We hypothesized that PI-IBS patient will show a more pro-inflammatory cytokine response, in this experimental *ex vivo* model.

## Material and Methods

### Study subjects

PI-IBS patients (n = 11) and healthy controls (n = 10) between the age of 18 and 75 years were enrolled in this study. The fecal and mucosal microbiota as well as the colon lymphocyte composition of some of the study participants were previously determined and reported [4]. All patients were recruited from the Gastroenterology Clinic, Örebro University Hospital between 2011 and 2012, met the Rome III diagnostic criteria for IBS at inclusion and positively confirmed onset of IBS symptoms after an episode of acute gastroenteritis with diarrhea and/or vomiting, which was confirmed by primary care medical records. Hence, these patients fulfilled the definition of PI-IBS [1]. Fecal culture at the onset of gastroenteritis was not performed in the majority of cases. Inclusion was limited to consecutive patients who experienced the initial gastroenteritis at least one year before study inclusion. None of the patients had a recurrent gastroenteritis between onset of disease and enrolment in the study. All but one of the PI-IBS patients had the diarrhea-predominant subtype of IBS (Table 1). Prior to study enrolment and according to established diagnostic criteria, microscopic colitis [17] and other inflammatory gut diseases were excluded by histologic screening of mucosal biopsies throughout the entire colon. Patients with signs of a systemic inflammatory condition were not included.

**Table 1:** Characterization [median (range)] of post-infectious irritable bowel syndrome patients (PI-IBS) and healthy controls (HC). Between-group comparisons of age and BMI were analyzed using two-tailed Student’s t-test and between-group comparisons of sex, bowel moments/day and pain score were analyzed with Mann-Whitney.

	PI-IBS	HC
	(n = 11)	(n = 10)
**Sex (F/M)**	7/4	5/5
**Age (years)**	40 (20–75)	26 (21–42)*
**BMI (kg/m** ^**2**^ **)**	24 (20–31)	24 (21–25)
**Bowel moments/day**	2.5 (2–3)	1 (0–2)**
**Pain score**	1 (0–3)	0 (0–0)**
**Duration of illness 0.5–1 / 1–5 / >5 (years)**	1/6/4	N/A
**Subtype (IBS-D/IBS-C/IBS-M)**	10/0/1	N/A

BMI = Body mass index. IBS-D = diarrhea-predominant IBS. IBS-C = constipation-predominant IBS. IBS-M = mixed IBS bowel pattern with both loose and hard stools. N/A = not applicable.

*p < 0*.05 = *,

*p < 0*.01 = **.

Exclusion criteria for both PI-IBS patients and healthy controls were intake of mesalamine, corticosteroids, antibiotics or immunosuppressive agents during the past year, current smoking, or excessive alcohol intake (>20 alcoholic consumptions per week). Participants refrained from medications likely to interfere with gastrointestinal function or pain and probiotics at least one week before endoscopic investigation. None of the participants had taken non-steroidal anti-inflammatory drugs (NSAIDs) during the last two weeks before entering the study. Pain and symptom scores were assessed in a three-scale questionnaire [4].

The healthy controls were recruited by advertisement at Örebro University. None of them had a self-reported history of chronic gastrointestinal complaints, chronic pain condition, infectious or inflammatory disorder, psychiatric illness, or were taking pharmaceutical agents. All subjects gave their written informed consent before participation. The study was performed in accordance with the principles of the declaration of Helsinki and was approved by the Regional Ethics Committee in Uppsala/Örebro (2010/261) and registered at ClinicalTrialgov NCT01787253.

### Intestinal tissue sampling

Distal colonoscopy was performed without prior bowel cleansing in the morning after an overnight fast. Colonic mucosal biopsies were obtained at a standardized location in the sigmoid colon, approximately 20–25 cm from the anal verge at the crossing with the *arteria iliaca communis*. Twelve biopsies (mean weight = 95 mg) were collected (Radical JawTM 4 Jumbo, Boston Scientific, USA) in cold phosphate buffered saline (PBS) with 5% bovine serum albumin (BSA) and were directly transferred to the lab.

### Mucosal biopsy stimulation

Biopsy specimens were transferred to sterile 12-well cell culture plates with a 15 mm net insert (Netwell Insert, 440 μm Polyester Mesh, Corning Incorporated, NY, USA) (epithelial layer up) into 500 μl pre-warmed 37°C RPMI-medium (1640 Medium. no Phenol Red, Invitrogen, Gibco, NY, USA) with 2.15% HEPES (Gibco, Paisle, Scotland, UK). A duplicate of biopsies was left unstimulated, while the others were stimulated in duplicates with a bacterial suspension containing a total of 10^6^ bacteria/well. The culture plate was incubated for 24 h in 37°C at 5% CO_2_. Only those biopsies that showed a cell viability of > 70% were included in the analyses.

### Bacterial strains and growth conditions for stimulation activity assays

The bacterial strains used for mucosal stimulation in this study were: *Ruminococcus gnavus* (CCUG 33437), *Bacteroides ovatus* (CCUG 4943 T), *Eubacterium limosum* (CCUG 16793-T), *Subdoligranum variabile* (CCUG 47106 T) and *Akkermansia muciniphila* (CCUG 64013 T). All strains were obtained from the Culture Collection, University of Gothenburg, Sweden. After revival, the strains were grown anaerobically in Fastidious Anaerobe Broth (Lab M, Lancashire, UK) supplemented with 10.0% (w/v) D-glucose (AnalaR Normapur, VWR International BVBA, Leuven, Belgium) at 37°C. 10^6^ bacterial cells per 500 μL HEPES medium were used for the stimulation.

### LUMINEX

The LUMINEX assay was performed according to the manufacture´s instructions MILLIPLEX MAP Kit (Human Cytokine / Chemokine 96-Well Plate Assay, 2009, Millipore Corporation, MA, USA). Briefly, eight different bead suspensions of antibodies, specifically directed against each of the eight cytokines (i.e. IL-1β, IL-2, IL-8, IL-10, IL-13, IL-17, TNF-α and IFN-γ, respectively), were irradiated with ultrasound and mixed in a solution. The 96-well filter plates were washed in RPMI (1640 medium without Phenol Red, Invitrogen, Gibco, NY, USA) with 1 M HEPES (Gibco, Paisley, Scotland, UK) at room temperature for 10 minutes while shaking. 25 *μ*l standards, controls and samples were added in duplicates, and diluted 1:1 with RPMI with 1 M HEPES. The cytokine-bead solution was added to each well and the plate was incubated while shaking at 4°C overnight. The plates were analyzed on the Luminex 200 ^TM^ (Luminex Corporation, Austin, Texas, USA) using the Xponent software (Version 3.1, Software Solution for Luminex, Hoorn, The Netherlands). Samples with < 10 beads were excluded from analysis. Mean of duplicate samples represented the cytokine response.

### Statistical analysis

Data analysis was carried out with the latest version (R.2.15.2) of R [18]. After log_2_-transformation data were approximately normally distributed. The associations between the cytokine and group (i.e. PI-IBS patients or healthy controls) after bacterial stimulation was calculated for each cytokine with linear mixed modeling. Estimates of variance and covariance in the parameters were taken into account by restricted maximum likelihood modeling (REML), and appropriate ANOVA type III SS run to test for significance of the fixed effects of disease/control, bacterial treatment, or their interaction. Spearman’s rank correlation coefficient was determined to assess correlations between cytokine concentrations and the subjects’ age. Mixed model results were considered significant if *p<0*.*05*.

## Results

There were no significant differences between PI-IBS patients and healthy controls regarding BMI or sex. However, the age between the PI-IBS patients and healthy controls differed significantly (*p < 0*.*05*). The secretion of IL-8 correlated significantly to age in healthy controls (r = -0.32; 95% CI = -0.06 to -0.54; *p < 0*.*05*), while the TNF- α secretion correlated significantly to age in PI-IBS as well as healthy controls (r = -0.25; 95% CI = -0.02 to -0.45; *p < 0*.*05*). No significant correlation between age and any of the other cytokines (i.e. IL-1β, IL-2, IL-10, IL-13, IL-17, or IFN-γ) was found.

**Fig 1:**
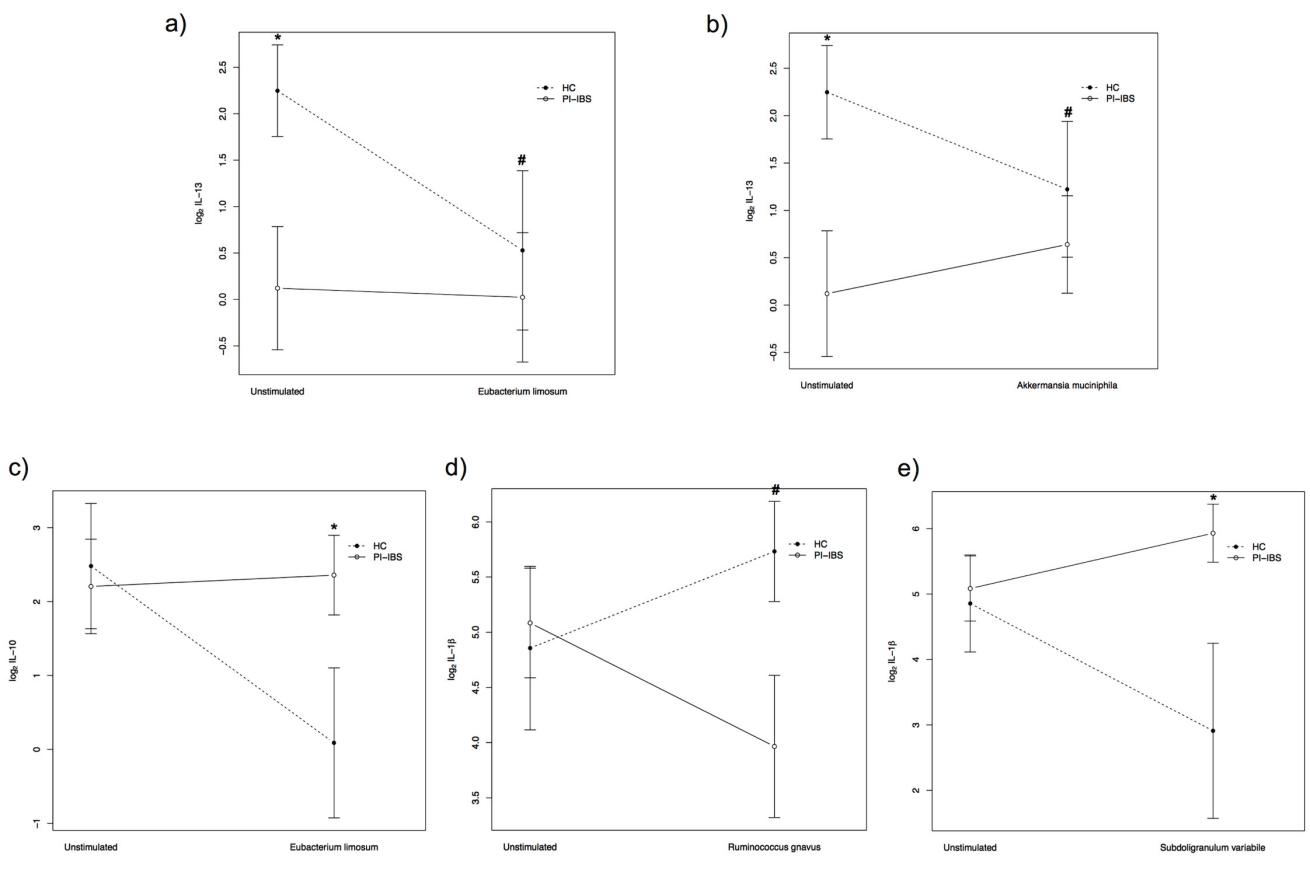
Cytokine release [Log_2_ Mean ± SE, pg mL^-1^] in the supernatant in post-infectious irritable bowel syndrome (PI-IBS) patients (n = 11) and healthy controls (HC) (n = 10) after bacterial stimulation of colonic biopsies ex vivo. a) Interleukin-13 (IL-13) release without bacterial stimulation and after stimulation with *Eubacterium limosum*. b) IL-13 release without bacterial stimulation and after stimulation with *Akkermansia muciniphila*. c) Interleukin-10 (IL-10) release without bacterial stimulation and after stimulation with *Eubacterium limosum*. d) Interleukin-1β (IL-1 β) release without bacterial stimulation and after stimulation with *Ruminococcus gnavus*. e) Interleukin-1β (IL-1 β) release without bacterial stimulation and after stimulation with *Subdoligranulum variabile*. * = p < 0.*05*, *# = p < 0*.*1*.

Baseline IL-13 release from biopsies without bacterial stimulation was significantly lower in PI-IBS patients compared to healthy controls (log_2_ fold change = 2.0, *p < 0*.*05*, Fig 1a). showed no significant difference The release of the other cytokines (i.e. IL-1β, IL-2, IL-8, IL-10, IL-17, TNF-α or IFN-γ) in biopsies without prior bacterial stimulation did not significantly differ between PI-IBS patients and healthy controls (Table 2).

**Table 2:** Cytokine levels [pg mL^-1^, ng mL^-1^ for IL-8] (mean ± SE), in supernatants of mucosal colonic biopsies after 24 h microbial stimulation. The biopsies were either unstimulated or stimulated with the bacterial strains *Bacteroides ovatus*, *Ruminococcus gnavus*, *Akkermansia muciniphila*, *Subdoligranulum variabile* or *Eubacterium limosum*, respectively. The significance of the differences was calculated between PI-IBS patients (n = 11) and HC (n = 10) and denoted with a * = p < 0.05, trends are shown in bold. Statistical significance was calculated on basis of log_2_ transformed data (see Statistical analysis).

	*Unstimulated*		*Bacteroides ovatus*		*Ruminococcus gnavus*		*Akkermansia muciniphila*		*Subdoligranulum variabile*		*Eubacterium limosum*	
	HC	PI-IBS	HC	PI-IBS	HC	PI-IBS	HC	PI-IBS	HC	PI-IBS	HC	PI-IBS
**IL-8**	8.9 ± 3.4	8.7 ± 3.1	7.9 ± 3.1	4.6 ± 1.3	5.8 ± 2.4	7.1 ± 3.0	4.0 ± 1.7	1.4 ± 3.8	6.0 ± 2.3	5.0 ± 1.5	4.5 ± 2.3	2.5 ± 6.5
**IL-10**	10.2 ± 4.8	9.3 ± 3.9	8.7 ± 2.6	22.2 ± 8.2	5.8 ± 1.5	13.5 ± 4.8	4.8 ± 1.5	14.0 ± 4.3	2.6 ± 0.5	7.8 ± 2.0	1.7 ± 0.5*	7.1 ± 1.7*
**IL-17**	4.1 ± 0.8	3.5 ± 0.7	3.2 ± 0.4	3.2 ± 0.5	2.9 ± 0.5	3.2 ± 0.5	3.6 ± 0.8	2.5 ± 0.3	3.0 ± 0.6	3.8 ± 0.7	2.9 ± 0.5	3.7 ± 0.8
**IL-1β**	41.0 ± 21.1	63.2 ± 17.8	57.4 ± 25.9	41.7 ± 10.0	46.4 ± 1.2*	43.8 ± 5.3*	25.2 ± 5.0	154.2 ± 1.0	20.2 ± 1.2*	95.2 ± 4.5*	85.1 ± 32.7	68.6 ± 8.9
**IL-2**	6.9 ± 2.2	3.5 ± 1.0	4.2 ± 1.0	3.5 ± 1.0	5.6 ± 1.3	4.1 ± 1.6	3.3 ± 0.6	5.8 ± 1.8	4.4 ± 0.9	5.5 ± 1.3	2.0 ± 0.5	3.1 ± 0.7
**IL-13**	6.9 ± 1.9*	2.7 ± 1.1*	4.1 ± 0.8	4.9 ± 1.9	6.4 ± 1.7	3.5 ± 1.0	3.4 ± 0.9	1.8 ± 0.3	2.4 ± 0.2	2.2 ± 0.9	1.4 ± 0.5	1.4 ± 0.5
**IFN-g**	5.5 ± 1.0	5.1 ± 1.0	5.1 ± 1.0	4.6 ± 0.7	5.8 ± 0.9	4.8 ± 0.9	5.1 ± 1.2	4.1 ± 0.7	5.1 ± 0.9	5.7 ± 1.4	6.6 ± 2.2	4.5 ± 1.3
**TNFα**	33.3 ± 15.4	41.5 ± 11.1	30.9 ± 11.8	40.8 ± 10.1	51.0 ± 23.8	33.9 ± 11.4	13.4 ± 4.2	39.7 ± 10.7	14.9 ± 5.1	40.2 ± 14.1	28.2 ± 20.4	24.4 ± 5.0

Footnote: Minimum detectable concentrations (pg/mL) for the assay according to the manufacturer are: IL-8 = 0.3; IL-10 = 0.5; IL-17 = 0.3; IL-1β = 0.7; IL-2 = 0.6; IL-13 = 0.9; IFN-γ = 0.3; TNFα = 0.1.

Stimulation with *A*. *muciniphila* did not result in any significant difference in cytokine release (i.e. IL-1β, IL-2, IL-8, IL-10, IL-17, TNF-α or IFN-γ) in PI-IBS patients compared to healthy controls (Table 2). However, stimulation with *A*. *muciniphila* showed a tendency to increased IL-13 release in PI-IBS patients compared to healthy controls (log_2_ fold change = 1.7, *p = 0*.*08*, Fig 1b). Stimulation with *E*. *limosum* resulted in a significant decrease of IL-10 release in healthy controls compared to PI-IBS patients (log_2_ fold change = 2.7, *p < 0*.*05*, Fig 1c) and a tendency to a decrease of IL-13 release in healthy controls compared to PI-IBS patients, respectively (log_2_ fold change = 1.7, *p = 0*.*07*, Fig 1a). Stimulation with *E*. *limosum* did not result in significant differences in the release of any of the other cytokines (i.e. IL-1β, IL-2, IL-8, IL-17, TNF-α or IFN-γ) in PI-IBS patients compared to healthy controls (Table 2). Stimulation with *R*. *gnavus* did not result in significant differences in the release of any of the cytokines (i.e. IL-2, IL-8, IL-10, IL-13, IL-17, TNF-α or IFN-γ) in PI-IBS patients compared to healthy controls (Table 2). However, after stimulation with *R*. *gnavus* the mucosal specimens in PI-IBS patients showed a tendency to decreased IL-1β release compared to healthy controls (log_2_ fold change = 2.0, *p = 0*.*07*, Fig 1d). Stimulation with *B*. *ovatus* did not result in significant differences in the release of any of the cytokines (i.e. IL-1β, IL-2, IL-8, IL-10, IL-13, IL-17, TNF-α or IFN-γ) in PI-IBS patients compared to healthy controls (Table 2). *S*. *variabile* stimulation resulted in a significantly increased IL-1β release in PI-IBS patients compared to healthy controls (log_2_ fold change = 2.6, *p < 0*.*05*, Fig 1e). Stimulation with *S*. *variabile* did not result in significant differences in the release of any of the other cytokines (i.e. IL-2, IL-8, IL-10, IL-17, TNF-α, or IFN-γ) in PI-IBS patients compared to healthy controls (Table 2).

## Discussion

Irritable bowel syndrome is one of the most frequently diagnosed intestinal disorders in the Western world [19]. A subset of IBS patients develop symptoms after exposure to an enteric infection (between 3% and 35%) and they are denoted as post-infectious IBS (PI-IBS) patients [20]. The underlying pathology of the PI-IBS comprises alterations along the gut-brain axis including low-grade inflammation [5] and aberrations in the intestinal microbiota [2, 4]. However, there is still a lack of well-designed experimental studies connecting aberrations in the mucosal immune system with those of the intestinal microbial community in PI-IBS patients. Hence, in this study we measured the cytokine response from whole endoscopic colonic biopsies after *ex vivo* stimulation with single key commensal bacterial strains in order to further elucidate the pathophysiology of PI-IBS.

Baseline release of the anti-inflammatory cytokine IL-13 without bacterial stimulation was significantly lower in mucosal specimens from PI-IBS patients compared to healthy controls (Fig 1a). This finding is in line with the concept of high inflammatory responses in PI-IBS, as reported previously [6]. Some of the commensal bacteria selected for stimulation in this study are strict anaerobes and were difficult to culture until recently. Hence, very little is known about their intestinal bio-functionality. The stimulation method presented in this study builds a model for the *in vivo* situation, with the limitation that some metabolic actions, such as short chain fatty acid (SCFA) production by bacteria as *S*. *variabile* are unlikely to occur. Most of the lose mucus needed for SCFA production is probably not conserved during the tissue incubation. Hence, we cannot exclude the possibility that the observed differences in cytokine release in biopsies from PI-IBS patients compared to healthy controls were due to other traits and properties of each specific bacterium.

*E*. *limosum* is able to produce butyrate, which has been suggested to reduce IBS symptoms [21] and is considered to play an important role in the maintenance of gut health [22]. Our results displayed a significantly reduced IL-10 release in PI-IBS patients compared to healthy controls (Fig 1c) and a tendency to a decreased IL-13 release (Fig 1a) after *E*. *limosum* stimulation, which indicates a down-regulation of the anti-inflammatory immune response. On the other hand, stimulation of lymphocytes from IBS patients and healthy controls with lipopolysaccharides has previously resulted in enhanced IL-13 and reduced IL-10 expression [23]. Hence, the different immune responses to commensal bacteria in PI-IBS patients and general IBS patients may be the result of alterations in their intestinal microbial composition [4]. Stimulation with *A*. *muciniphila*, a bacterium that is considered to have beneficial metabolic effects for the host [14], resulted only in a tendency to increased release of the anti-inflammatory cytokine IL-13 in PI-IBS patients compared to healthy controls (Fig 1b). This absence of significance in IL-13 release between PI-IBS patients and healthy controls after stimulation with *A*. *muciniphila* (Fig 1b) and *E*. *limosum* (Fig 1c) may be caused by insufficient statistical power.

Increased mast cell numbers, differences in mast cell localization and activation have been suggested to be important in the pathophysiology of PI-IBS [24]. PI-IBS patients responded with increased release of the pro-inflammatory cytokine IL-1β compared to healthy controls when stimulated with *S*. *variabile*. This altered immune reaction in PI-IBS may be a consequence of aberrations in numbers and activation of mast cells, which consequently elevate IL-1β cytokine response in the intestine [25]. In agreement with our findings, increased levels of IL-1β in serum and fecal samples have been reported in D-IBS as well as PI-IBS patients [26].

The differences in cytokine responses found between PI-IBS patients and healthy controls could either be attributed to differences in the numbers and proportions of mucosal lymphocyte subsets [6] and/or alterations in their intestinal microbiota composition [4]. The mucosal lymphocyte subsets of PI-IBS patients may also be more reactive to bacterial stimulation, independent of proportion. This is supported by findings indicating that peripheral αβ^+^ T lymphocytes of IBS patients, compared to healthy controls, expressed increased proportions of the activation molecule CD28 and decreased proportions of the chemokine receptor CCR5, after stimulation with pathogen-associated molecular patterns, [27]. A possible explanation for the differences in cytokine release of PI-IBS patients in comparison to healthy controls could be a differential expression of Toll-like receptors (TLRs) on immune cells, as IBS patients displayed altered expressions of intestinal TLR2, TLR4, TLR7, and TLR8 receptors [28–30].

We have previously reported that PI-IBS patients have a slightly altered mucosal microbiota composition compared to that of healthy controls [4] and this variation may have affected the results of the present study. For instance, the difference in baseline IL-13 response between PI-IBS patients and healthy controls (Fig 1a) could be a functional consequence of this different microbiota composition. This is of special relevance regarding the strains that were initially selected on the basis of differences in microbial abundance between PI-IBS and healthy controls (e.g. *B*. *ovatus*, *R*. *gnavus* and *E*. *limosum)*. On the other hand, the clinical importance of the response of a single cytokine expression, e.g. IL-13, should not be overestimated, but rather included in the general concept of inflammation in PI-IBS, as previously observed [4].

The number of PI-IBS patients enrolled in this study was limited, as PI-IBS patients comprise only a minor proportion of all IBS patients. Inclusion criteria were constrained to patients that did not have antibiotic treatment and to those who had experienced the gastrointestinal infection at least one year prior to study enrolment. Despite the low number of participants in this study, we were able to reveal distinct differences in mucosal cytokine release between PI-IBS patients and healthy controls. Fecal culture at the onset of gastroenteritis was not performed in the majority of cases and therefore the intestinal infection could have been caused by various bacterial species. We cannot exclude either that the initial infection was caused by a virus. Another limiting factor of this study is that the tissue specimens were obtained only from the distal colon, even though both the small and large intestine are affected in the pathophysiology of PI-IBS patients [31, 32]. We decided to abstain from bowel cleansing prior to colonoscopy as cleansing may alter the naturally occurring mucosa-associated microbiota [33]. In addition, the vast majority of microbes are inhabitants of the large intestine where most host-microbe interactions occur [34]. Further, the biopsies were grown under aerobic conditions, which may affect the viability of the anaerobic bacterial strains. Our further studies will include analysis of viability and survival of bacteria to determine whether the aerobic and anaerobic capacities of each strain have major effects on the cytokine expression levels. The 24-hour culture time was chosen after a pilot experiment as it provided enough time for protein synthesis while the cells in the biopsies maintained a high viability. Interestingly, in most cases the bacterial stimulations were associated with reduced levels of cytokine release in PI-IBS patients as well as healthy controls. Even though we cannot provide a clear explanation for this phenomenon, one might speculate that the bacterial strains chosen in this assay represent regulatory bacteria that predominantly repress inflammatory signals. Hence, it would be of interest to include a pathogenic microbe as a positive control for increased pro-inflammatory cytokine secretion in a future study.

The altered mucosal immune response to commensal bacteria in PI-IBS patients suggests that commensal bacteria that do not cause an inflammatory response in healthy subjects may induce mucosal inflammatory activity in PI-IBS patients [6]. A similar hypothesis has previously been presented for inflammatory bowel disease, were it has been suggested that patients have an exaggerated immune response to their own naturally occurring intestinal microbiota [35]. The reported differences in the mucosal immune responses in PI-IBS patients compared to healthy controls were bacterial strain specific. This supports the hypothesis that some commensal bacteria have a stronger immune-modulatory effect than others. The differences in microbe-related cytokine release may also be associated to IBS symptomatology, which previously has been reported for lactobacilli and bifidobacteria strains [36]. Our findings support further studies on probiotic treatment in PI-IBS patients using specific bacterial strains. In addition, our *ex vivo* model for bacterial stimulation could be used as a tool for exploring the immunological and biochemical actions of the intestinal mucosa that commensal and pathogenic bacteria might induce.

In conclusion, PI-IBS patients differ from healthy controls with regard to mucosal cytokine release of both pro- and anti-inflammatory cytokines after *ex vivo* stimulation with selected commensal bacteria. Further, our results confirm the hypothesis that an altered immune response to commensal bacteria in the colon may play a role in the pathogenesis of PI-IBS.

## References

[1] Spiller RC. Inflammation as a basis for functional GI disorders. Best Pract Res Clin Gastroenterol. 2004;18(4):641–61. 10.1016/j.bpg.2004.04.002 15324705

[2] Jalanka-Tuovinen J, Salojarvi J, Salonen A, Immonen O, Garsed K, Kelly FM, et al Faecal microbiota composition and host-microbe cross-talk following gastroenteritis and in postinfectious irritable bowel syndrome. Gut. 2014;63(11):1737–45. 10.1136/gutjnl-2013-305994 . 24310267

[3] Rinttilä T, Lyra A, Krogius-Kurikka L, Palva A. Real-time PCR analysis of enteric pathogens from fecal samples of irritable bowel syndrome subjects. Gut Pathog. 2011;3(1). 10.1186/1757-4749-3-6 21518462 PMC3111350

[4] Sundin J, Rangel I, Fuentes S, Heikamp-de Jong I, Hultgren-Hörnquist E, de Vos WM, et al Altered faecal and mucosal microbial composition in post- infectious irritable bowel syndrome patients correlates with mucosal lymphocyte phenotypes and psychological distress. Alimentary Pharmacology and Therapeutics. 2014;Epub ahead of print. 10.1111/apt.13055 25521822

[5] Spiller RC, Jenkins D, Thornley JP, Hebden JM, Wright T, Skinner M, et al Increased rectal mucosal enteroendocrine cells, T lymphocytes, and increased gut permeability following acute Campylobacter enteritis and in post-dysenteric irritable bowel syndrome. Gut. 2000;47(6):804–11. 10.1136/gut.47.6.804 11076879 PMC1728147

[6] Sundin J, Rangel I, Kumawat AK, Hultgren-Hörnquist E, Brummer R-J. Aberrant mucosal lymphocyte number and subsets in the colon of post-infectious IBS patients. Scan J Gastro. 2014;49(9):1068–75. 10.3109/00365521.2014.926982 24919810

[7] Cotton JA, Beatty JK, Buret AG. Host parasite interactions and pathophysiology in Giardia infections. Int J Parasitol. 2011;41(9):925–33. 10.1016/j.ijpara.2011.05.002 21683702

[8] Benckert J, Schmolka N, Kreschel C, Zoller MJ, Sturm A, Wiedenmann B, et al The majority of intestinal IgA+ and IgG+ plasmablasts in the human gut are antigen-specific. J Clin Invest. 2011;121(5):1946–55. 10.1172/JCI44447 21490392 PMC3083800

[9] Martínez C, González-Castro A, Vicario M, Santos J. Cellular and Molecular Basis of Intestinal Barrier Dysfunction in the Irritable Bowel Syndrome. Gut Liver. 2012;6(3):305–15. 10.5009/gnl.2012.6.3.305 22844557 PMC3404166

[10] Marshall JK, Thabane M, Garg AX, Clark W, Meddings J, Collins SM, et al Intestinal permeability in patients with irritable bowel syndrome after a waterborne outbreak of acute gastroenteritis in Walkerton, Ontario. Aliment Pharmacol Ther. 2004;20(11–12):1317–22. 10.1111/j.1365-2036.2004.02284.x 15606393

[11] Barbara G, Zecchi L, Barbaro R, Cremon C, Bellacosa L, Marcellini M, et al Mucosal permeability and immune activation as potential therapeutic targets of probiotics in irritable bowel syndrome. J Clin Gastroenterol. 2012;46:52–5. 10.1097/MCG.0b013e318264e918 22955358

[12] Degnan BA, Macfarlane S, Quigley ME, Macfarlane GT. Starch utilization by Bacteroides ovatus isolated from the human large intestine. Curr Microbiol. 1997;34(5):290–6. 10.1007/s002849900184 9099629

[13] Falk P, Hoskins LC, Larson G. Bacteria of the human intestinal microbiota produce glycosidases specific for lacto-series glycosphingolipids. J Biochem. 1990;108(3):466–74. 2277039 10.1093/oxfordjournals.jbchem.a123223

[14] Ouwerkerk JP, de Vos WM, Belzer C. Glycobiome: bacteria and mucus at the epithelial interface. Best Pract Res Clin Gastroenterol. 2013;27(1):25–38. 10.1016/j.bpg.2013.03.001 23768550

[15] Kanauchi O, Fukuda M, Matsumoto Y, Ishii S, Ozawa T, Shimizu M, et al Eubacterium limosum ameliorates experimental colitis and metabolite of microbe attenuates colonic inflammatory action with increase of mucosal integrity. World J Gastroenterol. 2006;12(7):1071–7 16534848 10.3748/wjg.v12.i7.1071 PMC4087899

[16] Louis P, Flint HJ. Diversity, metabolism and microbial ecology of butyrate-producing bacteria from the human large intestine. FEMS Microbiol Lett. 2009;294(1):1–8. 10.1111/j.1574-6968.2009.01514.x 19222573

[17] Kumawat AK, Strid H, Elgbratt K, Tysk C, Bohr J, Hultgren Hörnquist E. Microscopic colitis patients have increased proportions of Ki67(+) proliferating and CD45RO(+) active/memory CD8(+) and CD4(+)8(+) mucosal T cells. J Crohns Colitis. 2013;7(9):694–705. 10.1016/j.crohns.2012.08.014 22995775

[18] Computing FfS. R Development Core Team. Vinenna, Austria. 2014;ISBN 3-900051-07-0(URL: http://www.R-project.org).

[19] Quigley EM, Abdel-Hamid H, Barbara G, Bhatia SJ, Boeckxstaens G, De Giorgio R, et al A global perspective on irritable bowel syndrome: a consensus statement of the World Gastroenterology Organisation Summit Task Force on irritable bowel syndrome. J Clin Gastroenterol. 2012;46(5):356–66. 10.1097/MCG.0b013e318247157c 22499071

[20] Spiller R, Garsed K. Postinfectious irritable bowel syndrome. Gastroenterology. 2009;136(6):1979–88. 10.1053/j.gastro.2009.02.074 19457422

[21] Kanauchi O, Mitsuyama K, Komiyama Y, Yagi M, Andoh A, Sata M. Preventive effects of enzyme-treated rice fiber in a restraint stress-induced irritable bowel syndrome model. Int J Mol Med 2010;25(4):547–55. 10.3892/ijmm_00000376 20198303

[22] Hamer HM, Jonkers D, Venema K, Vanhoutvin S, Troost FJ, Brummer RJ. Review article: the role of butyrate on colonic function. Aliment Pharmacol Ther. 2008;27(2):104–19. 10.1111/j.1365-2036.2007.03562.x 17973645

[23] Kindt S, Van Oudenhove L, Broekaert D, Kasran A, Ceuppens JL, Bossuyt X, et al Immune dysfunction in patients with functional gastrointestinal disorders. Neurogastroenterol Motil. 2009;21(4):389–98. 10.1111/j.1365-2982.2008.01220.x 19126184

[24] Ortiz-Lucas M, Saz-Peiró P, Sebastián-Domingo JJ. Irritable bowel syndrome immune hypothesis. Part one: the role of lymphocytes and mast cells. Rev Esp Enferm Dig. 2010;102(11):637–47. 1130-0108/2010/102/11/637-647. 21142384 10.4321/s1130-01082010001100004

[25] Hamilton MJ, Sinnamon MJ, Lyng GD, Glickman JN, Wang X, Xing W, et al Essential role for mast cell tryptase in acute experimental colitis. Proc Natl Acad Sci U S A. 2011;108(1):290–5. 10.1073/pnas.1005758108 21173247 PMC3017166

[26] Darkoh C, Comer L, Zewdie G, Harold S, Snyder N, Dupont HL. Chemotactic chemokines are important in the pathogenesis of irritable bowel syndrome. PLoS One. 2014;9(3). 10.1371/journal.pone.0093144 PMC3965506 24667736

[27] Rodríguez-Fandiño O, Hernández-Ruíz J, López-Vidal Y, Charúa L, Bandeh-Moghaddam H, Minzoni A, et al Intestinal recruiting and activation profiles in peripheral blood mononuclear cells in response to pathogen-associated molecular patterns stimulation in patients with IBS. Neurogastroenterol Motil. 2013;25(11):872–e699. 10.1111/nmo.12204 23937411

[28] Brint EK, MacSharry J, Fanning A, Shanahan F, Quigley EM. Differential expression of toll-like receptors in patients with irritable bowel syndrome. Am J Gastroenterol. 2011;106(2):329–3. 10.1038/ajg.2010.438 21102570

[29] Belmonte L, Beutheu-Youmba S, Bertiaux-Vandaële N, Antonietti M, Lecleire S, Zalar A, et al Role of toll like receptors in irritable bowel syndrome: differential mucosal immune activation according to the disease subtype. PLoS One. 2012;7(8):e42777 23028414 10.1371/journal.pone.0042777 PMC3461726

[30] McKernan DP, Gaszner G, Quigley EM, Cryan JF, Dinan TG. Altered peripheral toll-like receptor responses in the irritable bowel syndrome. Aliment Pharmacol Ther. 2011;33(9):1045–52. 10.1111/j.1365-2036.2011.04624.x 21453321

[31] Guilarte M, Santos J, de Torres I, Alonso C, Vicario M, Ramos L, et al Diarrhoea-predominant IBS patients show mast cell activation and hyperplasia in the jejunum. Gut. 2007;56(2):203–9. 17005763 10.1136/gut.2006.100594 PMC1856785

[32] Dunlop SP, Hebden J, Campbell E, Naesdal J, Olbe L, Perkins AC, et al Abnormal intestinal permeability in subgroups of diarrhea-predominant irritable bowel syndromes. Am J Gastroenterol. 2006;101(6):1288–94. 16771951 10.1111/j.1572-0241.2006.00672.x

[33] Harrell L, Wang Y, Antonopoulos D, Young V, Lichtenstein L, Huang Y, et al Standard colonic lavage alters the natural state of mucosal-associated microbiota in the human colon. PLoS One. 2012;7(2):e32545 10.1371/journal.pone.0032545 22389708 PMC3289660

[34] Brown EM, Sadarangani M, Finlay BB. The role of the immune system in governing host-microbe interactions in the intestine. Nat Immunol. 2013;14(7):660–7. 10.1038/ni.2611 23778793

[35] Matsuoka K, Kanai T. The gut microbiota and inflammatory bowel disease. Semin Immunopathol. 2014;11 25([Epub ahead of print]). 10.1007/s00281-014-0454-4 PMC4281375 25420450

[36] O'Mahony L, McCarthy J, Kelly P, Hurley G, Luo F, Chen K, et al Lactobacillus and bifidobacterium in irritable bowel syndrome: symptom responses and relationship to cytokine profiles. Gastroenterology. 2005;128(3):541–51. 10.1053/j.gastro.2004.11.050 15765388

